# The bacterial microbiome modulates the initiation of brain metastasis by impacting the gut-to-brain axis

**DOI:** 10.1016/j.isci.2025.111874

**Published:** 2025-01-22

**Authors:** Matteo Massara, Michelle Ballabio, Bastien Dolfi, Golnaz Morad, Vladimir Wischnewski, Eleni Lamprou, Joao Lourenco, Stéphanie Claudinot, Hector Gallart-Ayala, Rui Santalla Méndez, Annamaria Kauzlaric, Nadine Fournier, Ashish V. Damania, Matthew C. Wong, Julijana Ivanisevic, Nadim J. Ajami, Jennifer A. Wargo, Johanna A. Joyce

**Affiliations:** 1 Department of Oncology, University of Lausanne, 1011 Lausanne, Switzerland; 2 Ludwig Institute for Cancer Research, University of Lausanne, 1011 Lausanne, Switzerland; 3 Agora Cancer Research Centre Lausanne, 1011 Lausanne, Switzerland; 4 L. Lundin and Family Brain Tumor Research Center, Departments of Oncology and Clinical Neurosciences, Centre Hospitalier Universitaire Vaudois, 1011 Lausanne, Switzerland; 5 Department of Surgical Oncology, The University of Texas MD Anderson Cancer Center, Houston, TX 77054, USA; 6 Translational Data Science Facility, Swiss Institute of Bioinformatics, AGORA Cancer Research Centre, 1011 Lausanne, Switzerland; 7 Germ-free facility, Centre Hospitalier Universitaire Vaudois and University of Lausanne, 1066 Epalinges, Switzerland; 8 Metabolomics Unit, Faculty of Biology and Medicine, University of Lausanne, Lausanne, Switzerland; 9 Platform for Innovative Microbiome & Translational Research (PRIME-TR), Moon Shots™, Houston, TX 77054, USA; 10 Department of Genomic Medicine, The University of Texas MD Anderson Cancer Center, Houston, TX 77054, USA

**Keywords:** Microenvironment, Microbiome, Cancer

## Abstract

Brain metastases (BrMs) are the most common brain tumors in patients and are associated with poor prognosis. Investigating the systemic and environmental factors regulating BrM biology represents an important strategy to develop effective treatments. Toward this goal, we explored the contribution of the gut microbiome to BrM development by using *in vivo* breast-BrM models under germ-free conditions or antibiotic treatment. This revealed a detrimental role of gut microbiota in fostering BrM initiation. We thus evaluated the impact of antibiotics and BrM outgrowth on the gut-brain axis. We found the bacterial genus *Alistipes* was differentially present under antibiotic treatment and BrM progression. In parallel, we quantified circulating metabolites, revealing kynurenic acid as a differentially abundant molecule that impaired the interaction between cancer cells and the brain vasculature in *ex vivo* functional assays. Together, these results illuminate the potential role of gut microbiota in modulating breast-BrM via the gut-to-brain axis.

## Highlights

• Depletion of gut bacteria delays BrM onset in a breast-BrM model *in vivo*
• *Alistipes* bacteria in the gut are differentially altered by antibiotics and at BrM endpoint
• Kynurenic acid impedes cancer cell-brain vessel interactions *ex vivo*

## Graphical abstract

**Figure:**
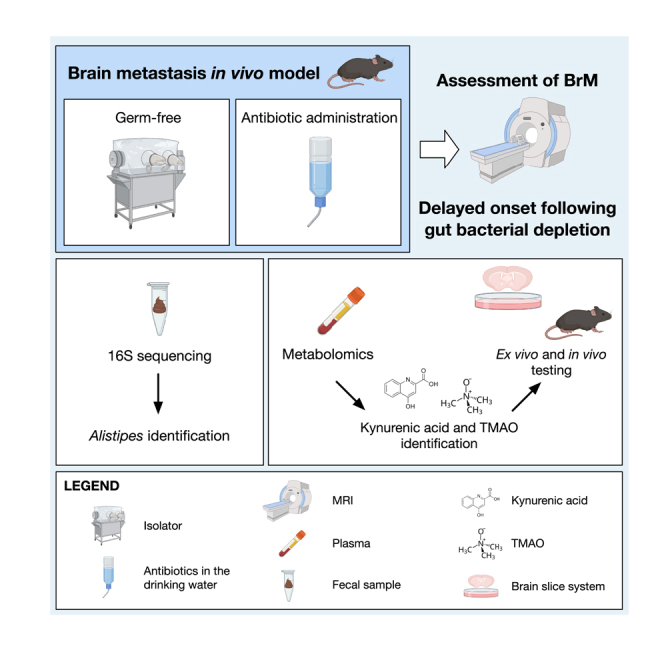

## Introduction

The development of brain metastasis (BrM), originating primarily from lung cancers (40%–60%), breast cancer (15%–25%), and melanoma (5–20%), represents a formidable clinical challenge. The limited efficacy of current treatment options results in patient survival rates that are often measured by just a few months following BrM diagnosis.^1^^,^^2^ To address this challenge, new approaches are urgently needed. One promising strategy centers on investigating and targeting the tumor microenvironment (TME), which is recognized as a key regulator of metastatic dissemination and outgrowth.^3^^,^^4^ The brain TME is distinguished by several unique cell types, including astrocytes, neurons, and microglia; an immunosuppressive environment; and the presence of the blood-brain barrier (BBB), which can interfere with effective drug delivery to the brain.^5^

The complex interplay between the tumor and its microenvironment is influenced by both cancer-driven factors as well as patient-intrinsic variables such as the microbiome.^4^ Investigating how systemic factors regulate BrM biology represents a potentially targetable strategy for developing effective treatments for brain tumors.^6^ Indeed, the brain is in bidirectional communication with the gut microbiome, creating the gut-to-brain axis.^7^ Bacteria in the gut can impact the central nervous system (CNS) by regulating the abundance of metabolites and neurotransmitters.^8^ In turn, the CNS can regulate peristalsis via the vagus nerve activity.^8^ Several non-cancer-related brain pathologies have been reported to impact the gut-to-brain axis, which may then exacerbate disease progression.^9^ In cancer, the host-associated polymorphic microbiome has been recently added as an enabling characteristic to the canonical hallmarks of cancer, in recognition of the broad impact of the microbiome on carcinogenesis, tumor outgrowth, and regulation of therapeutic efficacy.^10^

The gut-brain axis can also be impacted in gliomas.^11^^,^^12^ An altered gut microbiome composition has been reported in patients with primary brain tumors.^13^^,^^14^ In mouse glioma models, systemic antibiotic treatment promoted tumor outgrowth,^15^^,^^16^ in association with increased FoxP3-positive regulatory T cells in the TME.^16^ Moreover, in a mouse glioma model engrafted with a humanized microbiome, the bacterial species *Bacteroides cellulosilyticus* was associated with enhanced anti-PD-1 treatment efficacy.^17^ Finally, bacteria have also been reported to be an integral part of the TME in human gliomas,^18^ although their role has yet to be fully elucidated. In this study, we sought to specifically assess the role of the bacterial microbiome in the biology of metastatic brain cancer, taking advantage of *in vivo* breast-BrM models to investigate the gut-to-brain axis.

## Results

### Complete bacteria depletion delays brain metastasis initiation in a breast-BrM model

**Figure 1:**
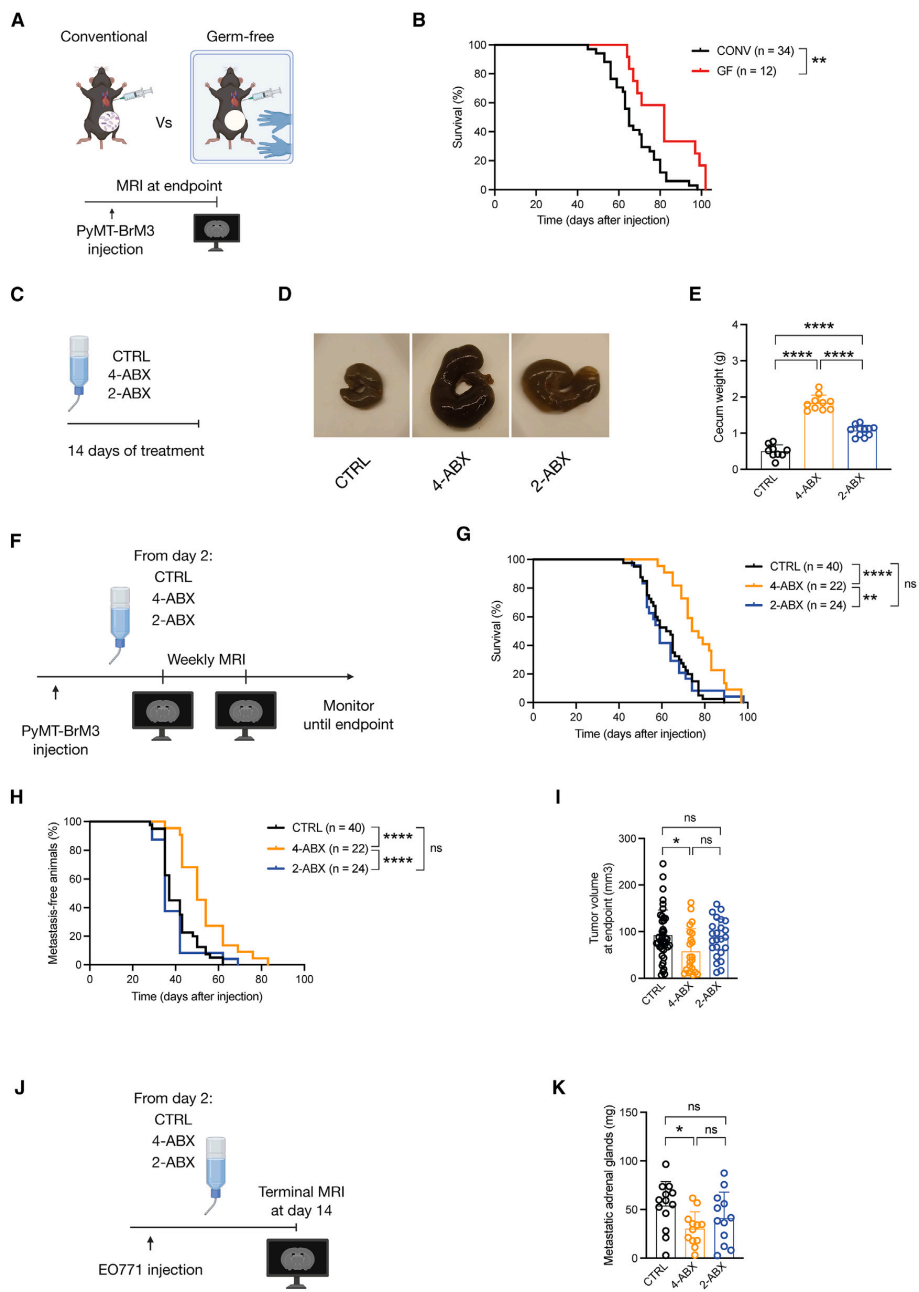
Bacterial microbiome manipulation delays breast metastasis (A) Experimental schematic for the investigation of the microbiome in preclinical breast-BrM models. Immunocompetent mice were housed in either conventional (CONV) or germ-free (GF) conditions and intracardially injected with PyMT-BrM3 cells. Mice were monitored throughout, and sacrificed at the experimental endpoint, after a single MRI was performed to assess BrM outgrowth. (B) Kaplan-Meier curves showing the overall survival of mice in CONV and GF conditions. *n* = 34 for CONV, *n* = 12 for GF; sum of three independent experiments. (C) Experimental schematic for the collection of cecum samples from healthy mice treated with antibiotics. Mice received either 4-ABX, 2-ABX, or control treatments in the drinking water for 14 days. (D) Representative images of ceca following CTRL, 4-ABX, or 2-ABX administration to healthy mice for 14 days. (E) Analysis of cecum weight following CTRL, 4-ABX, or 2-ABX administration to healthy mice for 14 days *n* = 9 for CTRL, *n* = 10 for 4-ABX, *n* = 11 for 2-ABX. (F) Experimental schematic for antibiotic administration in breast-BrM. Mice were intracardially injected with PyMT-BrM3 cells and 2 days later received either 4-ABX, 2-ABX, or control treatments in the drinking water, which was maintained until the end of the experiment. Mice were monitored by regular MRI to assess BrM outgrowth. (G and H) Kaplan-Meier curves showing (G) the overall survival and (H) the BrM onset of CTRL, 4-ABX-treated, and 2-ABX-treated mice, *n* = 40 for CTRL, *n* = 22 for 4-ABX, *n* = 24 for 2-ABX; sum of three independent experiments. (I) Tumor volume at the endpoint of mice in the CTRL, 4-ABX, or 2-ABX groups. For (G, H, and I), *n* = 40 for CTRL, *n* = 22 for 4-ABX, and *n* = 24 for 2-ABX; sum of three independent experiments. (J) Experimental schematic for the antibiotic administration in the EO771 model. Immunocompetent mice were intracardially injected with EO771 cells, and 2 days later either 4-ABX, 2-ABX, or control treatments were administered in the drinking water and maintained until the end of the experiment. Mice were sacrificed 14 days after the cancer cell injection and imaged by MRI at the endpoint to assess BrM outgrowth. (K) Metastatic adrenal gland weight at the endpoint in the EO771 model treated with CTRL, 4-ABX, or 2-ABX. Each data point represents the sum of two adrenal glands from a single mouse. *n* = 13 for CTRL, *n* = 12 for 4-ABX, *n* = 12 for 2-ABX; single experiment. Statistical analysis in (B, G, H) was performed using the Mantel-Cox log rank test. Statistical analysis in (E and I) was performed using one-way ANOVA. Statistical analysis in (K) was performed using Kruskal-Wallis test. Data are represented as mean ± SD. ∗*p* < 0.05; ∗∗*p* < 0.01; ∗∗∗∗*p* < 0.0001; ns: non-significant.

To examine the functional role of the bacterial microbiome in BrM biology, we employed an *in vivo* breast-BrM model utilizing the PyMT-BrM3 breast cancer cell line, which exhibits enhanced brain-homing capacity.^19^ We injected these cells into mice housed either in a germ-free (GF) facility or under conventional conditions (CONV), in parallel. Mice were closely monitored and sacrificed at the designated endpoint following a single terminal MRI to calculate BrM tumor volume (Figure 1A). Interestingly, mice housed in the GF facility displayed overall prolonged survival compared to their conventionally housed counterparts (Figure 1B). At the experimental endpoint, however, no evident differences were detected in BrM number (Figure S1A) or volume (Figure S1B), indicating similar metastatic outgrowth under both conditions despite the initial increased survival in the GF group.

We next performed a complementary series of experiments to deplete bacteria, via the administration of antibiotics in the drinking water to conventionally housed mice, utilizing the same breast-BrM model. Mice received either a broad-spectrum antibiotic mix (4-ABX: Ampicillin, Metronidazole, Neomycin, Vancomycin) commonly used to deplete the bacterial microbiome in pre-clinical studies^20^ or a more selective antibiotic mix (2-ABX: Bacitracin, Neomycin), which has been reported to not be absorbed by the gut,^21^ in the drinking water or control (CTRL) (2% sucrose for all groups). We first evaluated the effect of the two different antibiotic cocktails by treating healthy, non-tumor-bearing mice over a 14-day period (Figure 1C). Both the 4-ABX and 2-ABX groups exhibited increased cecum weight compared to CTRL, with the most pronounced effect observed in the 4-ABX group (Figures 1D and 1E), indicative of the induction of gut dysbiosis following antibiotic administration.^22^

We subsequently initiated the breast-BrM model in independent cohorts by intracardially injecting mice with the PyMT-BrM3 cancer cell line, and antibiotic treatment was started 2 days later, continuing until the trial endpoint, incorporating weekly MRI of the mice (Figures 1F and S1C). We designed this experiment with the goal of mimicking a potential translational setting in which antibiotics are administered orally at the time when cancer cells may be extravasating in the brain, as observed in our previous publication.^23^ Notably, an increase in survival was observed only in mice treated with the 4-ABX cocktail but not with the 2-ABX (Figure 1G). The 4-ABX data thus phenocopy the results obtained in the GF setting (Figure 1B). To determine whether the prolonged survival following antibiotic administration was the consequence of either altered metastatic seeding or tumor outgrowth in the brain, we assessed BrM onset, defined as the first day of BrM detection by MRI. We then classified BrM progression as the difference in time between BrM onset and the disease endpoint. Interestingly, mice treated with the 4-ABX cocktail exhibited delayed BrM onset, whereas no difference was detected in mice treated with the 2-ABX cocktail compared to controls (Figure 1H). No significant differences were observed in BrM progression (Figure S1D), nor in the number of BrM lesions (Figure S1E), in either treatment group compared to controls. By contrast, we observed a significant overall reduction in tumor volume at the endpoint in the 4-ABX group (Figure 1I). We also investigated the immune cell landscape of established BrM lesions, finding no difference in the relative abundance of the major immune cell populations infiltrating the tumor (Figure S1F). These results are consistent with our conclusion that bacterial microbiome manipulation impacts BrM onset uniquely and not the subsequent tumor outgrowth.

To extend these findings, we evaluated an additional breast-BrM model by intracardially injecting the EO771 breast cancer cell line in mice and administering antibiotics starting 2 days later until the endpoint. Mice were sacrificed 14 days after cancer cell injection due to weight loss, and a terminal MRI was performed to assess BrM outgrowth (Figure 1J). We observed a trend toward reduced BrM penetrance in mice treated with 4-ABX compared to controls but not for the 2-ABX group (Figure S1G). Additionally, we evaluated the mass of metastatic adrenal glands and ovaries, which represent the two major extracranial metastatic sites in the EO771 model. We observed a significant reduction in the mass of metastatic adrenal glands after 4-ABX administration, but not 2-ABX treatment (Figure 1K), and no differences in the mass of metastatic ovaries following either ABX treatment group (Figure S1H). In sum, our results from the GF and ABX experiments together indicate that the gut microbiome can modulate the initial steps of breast metastasis establishment in the brain, as well as the adrenal glands.

### Evaluation of the gut microbiome following antibiotic administration and at breast-BrM endpoint

**Figure 2:**
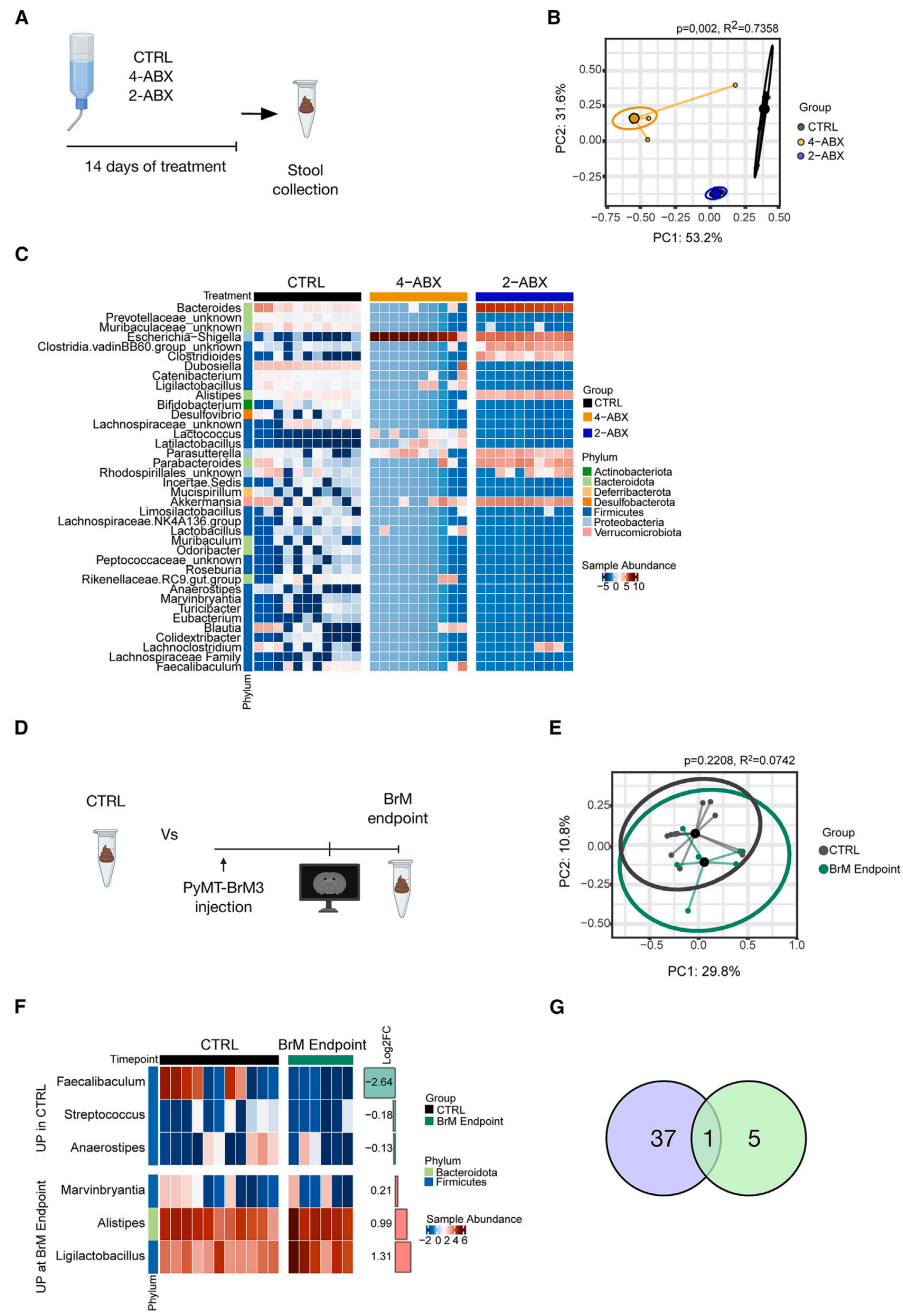
Gut bacteria composition is impacted by antibiotics and at the BrM endpoint (A) Experimental schematic for the collection of feces following administration of CTRL, 4-ABX, or 2-ABX to healthy mice for 14 days via the drinking water. (B) Beta-diversity analysis of gut bacterial composition in healthy mice treated with either CTRL, 4-ABX, or 2-ABX, assessed at the bacterial genus level. (C) Heatmap showing relative abundance of gut bacteria at the genus level following CTRL, 4-ABX, or 2-ABX administration to healthy mice for 14 days. Only genera with q < 0.05 are displayed. Sample sizes in (B, C), *n* = 11 for CTRL, *n* = 10 for both 4-ABX and 2-ABX groups. (D) Experimental schematic for feces collection from healthy CTRL and BrM-endpoint mice. (E) Beta-diversity analysis of gut bacterial composition from CTRL and BrM-endpoint mice at the bacterial genus level. (F) Heatmap showing relative abundance of gut bacteria at the genus level in CTRL vs. BrM-endpoint mice. Only genera with q < 0.05 are shown. For (E, F), *n* = 11 for CTRL; *n* = 6 for BrM endpoint. (G) Venn diagram showing numbers of differentially abundant bacterial genera after 2-ABX and 4-ABX treatments (blue circle) and at BrM-endpoint (green circle). In the intersected area is included the genus *Alistipes*. Statistical analysis for (B and E) was performed using Adonis2 test. Statistical analysis in (C) was performed using the Kruskal-Wallis test, and analysis in (F) was performed using the Analysis of Compositions of Microbiomes with Bias Correction (ANCOM-BC).

Given the impact of the gut microbiome on BrM initiation shown above (Figure 1H), we next investigated how the gut-to-brain axis might be altered in the PyMT-BrM3 model. We thus evaluated the impact of both antibiotic regimens and BrM outgrowth on bacterial composition in the gut. Fecal samples were collected from healthy mice treated with either of the two antibiotic formulations (4-ABX, 2-ABX) or controls for 14 days, and the bacterial microbiome composition was assessed by 16S rRNA gene sequencing (Figure 2A) (Table S1). As expected, we observed a pronounced difference in the overall gut bacteria community in mice treated with the two antibiotic cocktails compared to controls, as measured by beta diversity (Figure 2B). Specifically, 4-ABX administration led to a significant reduction in major commensal gut bacteria (Figure 2C). In fecal samples from the 2-ABX group, we noted a significant reduction in the abundance of gut bacterial taxa, though to a lesser extent by comparison to the 4-ABX treatment group (Figure 2C), as also indicated by the Shannon diversity index (Figure S2A). This is consistent with the intermediate increase in cecum weight in the 2-ABX group compared with the CTRL and 4-ABX cohorts (Figure 1E).

Concurrently, using 16S rRNA gene sequencing, we compared the gut microbiome composition between healthy control mice (same group as analyzed in Figures 2A–2C) and BrM-bearing mice at endpoint (Figure 2D) (Table S1). Interestingly, we observed an overall similar gut bacterial microbiome composition in both groups, healthy control versus BrM-endpoint (CTRL group) (Figures 2E and S2B), indicating a modest impact of PyMT-BrM3-derived breast-BrM outgrowth on the gut bacterial microbiome. Nonetheless, significant differences in the abundance of six specific genera were evident (Figure 2F). We next sought to identify which bacterial population might potentially influence BrM initiation. To this end, we intersected the results of the two sequencing analyses (Figures 2C and 2F), which revealed *Alistipes* as the sole bacterial genus (Figure 2G) altered both at the BrM endpoint (enriched) and also by 4-ABX administration (depleted).

### Evaluation of the circulating metabolites after antibiotic administration and in the breast-BrM endpoint

**Figure 3:**
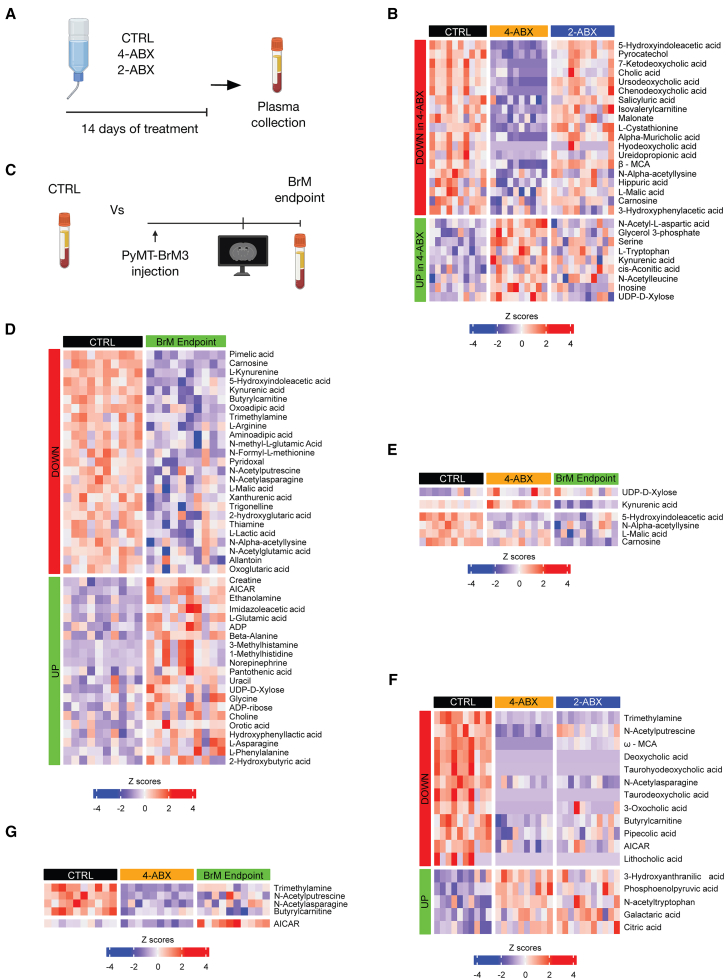
Abundance of circulating metabolites is impacted by antibiotics and at the BrM endpoint (A) Experimental schematic for plasma collection following CTRL, 4-ABX, or 2-ABX administration to healthy mice for 14 days in the drinking water. (B) Heatmap representation of plasma metabolites exhibiting differential abundance in CTRL vs. 4-ABX, but not in CTRL vs. 2-ABX as determined by an ANOVA test. *n* = 10 for both CTRL and 4-ABX groups; *n* = 11 for 2-ABX. (C) Experimental schematic for plasma collection from healthy CTRL and BrM-endpoint mice. (D) Heatmap showing plasma metabolites differentially abundant in CTRL vs. BrM endpoint. *n* = 10 for both CTRL and BrM endpoint cohorts. (E) Heatmap representation of plasma metabolites presented in Figure S3A. (F) Heatmap illustrating plasma metabolites exhibiting differential abundance in CTRL vs. 4-ABX and 2-ABX groups, as determined by an ANOVA test. *n* = 10 for both CTRL and 4-ABX groups; *n* = 11 for 2-ABX. (G) Heatmap representation of plasma metabolites shown in Figure S3B.

We next investigated the systemic impact of the different antibiotic formulations and BrM outgrowth, aiming to identify potential connections between alterations in the gut bacterial microbiome and delayed BrM onset. We focused on circulating metabolites, functional molecules that are produced and processed by gut bacteria that can impact cancer, specifically polar metabolites and bile acids.^24^ We collected plasma from healthy non-tumor-bearing animals treated for 14 days with either 4-ABX, 2-ABX, or CTRL (Figure 3A) and performed a differential metabolite analysis. We first compiled a list of the metabolites altered by both antibiotic treatments (Table S2). Given the delayed BrM onset only in the 4-ABX treatment group and not in the 2-ABX group (Figure 1H), we then further refined our analysis to identify statistically different molecules altered specifically in the 4-ABX treatment group and not in the 2-ABX group compared to controls. This resulted in a subset of 28 molecules whose levels were either increased or depleted in the 4-ABX treatment group (Figure 3B).

Concurrently, we also compared circulating metabolites from BrM-bearing mice (CTRL group) at endpoint versus healthy control mice (the same group as analyzed in Figures 3A and 3B) to investigate the systemic impact of metabolites caused by BrM (Figure 3C). Following normalization and statistical analysis, we identified a group of 46 molecules as differentially abundant at the BrM-endpoint compared to non-tumor-bearing control mice (Figure 3D). Intersection of the results of the two metabolomics analyses (Figures 3B and 3D) resulted in a list of six molecules (Figure S3A). Among these, kynurenic acid (KYNA) was the only metabolite that accumulated in the 4-ABX group and which was depleted conversely at the BrM endpoint in control mice (Figure 3E). KYNA, a metabolite derived from L-tryptophan, is known for its neuroprotective properties, exerting its effects by blocking N-methyl-D-aspartate (NMDAR) receptors.^25^ Given the regulation of KYNA by the bacterial microbiome and its described neuroprotective role, we hypothesized that KYNA may have a functional role in regulating BrM biology in our model. Additionally, we investigated circulating metabolites affected by both 4-ABX and 2-ABX treatments, detecting 12 molecules of lower abundance and 5 of higher abundance (Figure 3F). The intersection of this analysis with the list of metabolites altered in BrM-bearing mice at the endpoint (Figure 3D) resulted in a refined list of five molecules (Figure S3B), including trimethylamine (Figure 3G). Notably, the liver-processed metabolite trimethylamine n-oxide (TMAO) has been reported to regulate the immune response during primary breast cancer outgrowth.^26^ Thus, we focused on KYNA and TMAO as two metabolites potentially involved in BrM biology whose effects were previously described in neurobiology and breast cancer progression, respectively.

### KYNA is a metabolite partially involved in modulating brain metastasis seeding

**Figure 4:**
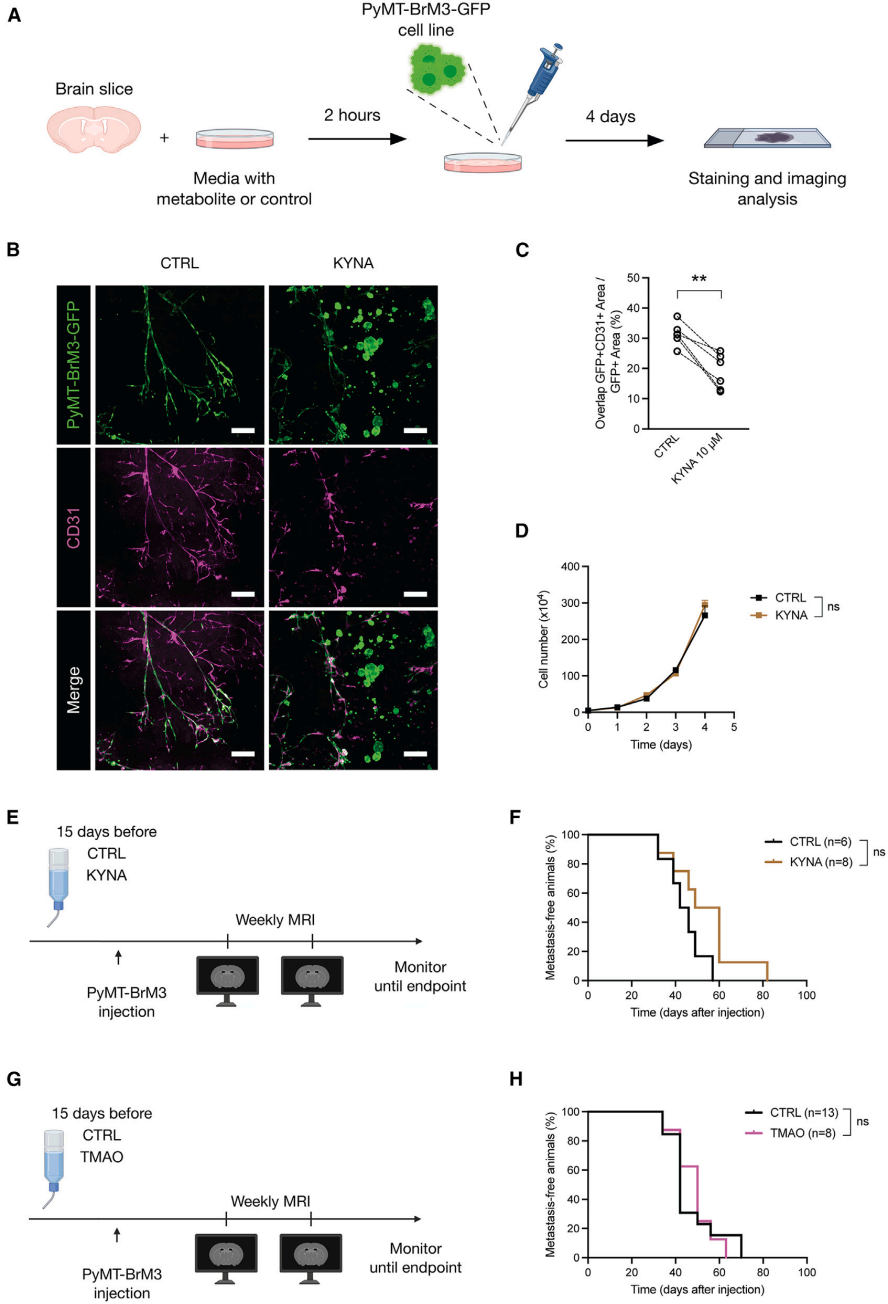
Kynurenic acid interferes with BrM cell-vessel interactions in *ex vivo* brain assays (A) Schematic representation of the brain slice co-culture experiment. Brain slices obtained from healthy mice were incubated for 2 h with kynurenic acid (KYNA) or vehicle (CTRL) before being co-cultured with PyMT-BrM3-GFP cells. Following a 4-day incubation period, slices were evaluated by immunofluorescence (IF) staining. (B) Representative IF images of PyMT-BrM3 cells (green) and CD31 (magenta) in brain slices that were incubated for 4 days with CTRL or KYNA. Scale bar: 200 μm. (C) Quantification of tissue area co-stained for both GFP and CD31 as a proportion of the total GFP+ area. *n* = 6 mice. Sum of two independent experiments. (D) Growth curve of PyMT-BrM3 cells *in vitro* incubated in the presence of KYNA or CTRL. *n* = 4 per time point. Experiment was repeated twice; sum of two independent experiments. (E) Experimental schematic for the KYNA administration in breast-BrM. Mice received KYNA or control treatments in the drinking water 15 days prior to intracardial injection of PyMT-BrM3 cells and were maintained on this treatment until the end of the experiment. Mice were monitored by regular MRI to assess BrM outgrowth. (F) Kaplan-Meier curves showing the BrM onset of CTRL and KYNA treated mice. *n* = 6 for CTRL, *n* = 8 for KYNA; single experiment. (G) Experimental schematic for the TMAO administration in breast-BrM. Mice received TMAO or control treatments in the drinking water 15 days prior to intracardial injection of PyMT-BrM3 cells and were maintained on this treatment until the end of the experiment. Mice were monitored by regular MRI to assess BrM outgrowth. (H) Kaplan-Meier curves showing the BrM onset of CTRL and TMAO treated mice. *n* = 13 for CTRL, *n* = 8 for TMAO; single experiment. Statistical analysis in (C) was performed using paired t test. Statistical analysis in (D) was performed using two-way ANOVA. Statistical analysis in (F and H) was performed using the Mantel-Cox log rank test. Data are represented as mean ± SD. ∗∗*p* < 0.01; ns: non-significant.

To investigate whether these specific metabolites might regulate BrM seeding, we assessed the interaction between cancer cells and brain blood vessels using an *ex vivo* model, representing the initial step of metastasis colonization in the brain.^27^ Healthy mouse brain slices were generated and incubated with PyMT-BrM3-GFP-labeled cancer cells for 4 days in the presence of either KYNA or the vehicle control (Figure 4A). In the control group, we observed that cancer cells tightly covered CD31^+^ vessels (Figure 4B), consistent with observations from *in vivo* studies using intravital microscopy in a different BrM model^28^ and our own results from confocal microscopy analyses in this breast-BrM model.^23^ Intriguingly, KYNA treatment significantly reduced the cancer cell-blood vessel interaction (Figures 4B and 4C), resulting in vessel-independent cancer cell islets (Figure 4B). This may potentially explain the effect on BrM initiation *in vivo* (under 4-ABX treatment), by interfering with the BrM cell-vessel interaction, which represents a critical step in brain colonization by circulating cancer cells.^29^ Importantly, no difference in viability was found when KYNA was added to cancer cells alone *in vitro* (Figure 4D), indicating a specific role for KYNA in modulating the cancer cell-brain vessel interaction during BrM onset. Notably, the increased abundance of systemic KYNA by antibiotic administration, and its reduction at the BrM endpoint, is associated with differential abundance of the genus *Alistipes*, one of the bacterial taxonomies altered by antibiotic administration and at the BrM endpoint (Figure 2G). To evaluate the *in vivo* relevance of our finding regarding the effect of KYNA supplementation *ex vivo*, we performed an experiment administering KYNA in the drinking water of mice starting 2 weeks prior to cancer cell injection and continuing for the entire duration of the experiment until the endpoint (Figure 4E). We observed a trend, though not significant, in delaying BrM onset compared to controls (Figure 4F). We found no difference in terms of overall survival (Figure S4A), BrM progression (Figure S4B), BrM number (Figure S4C), and volume (Figure S4D), indicating only a partial phenocopy of the delayed BrM onset observed upon 4-ABX administration (Figure 1H), suggesting multiple additional factors contributing to the phenotype.

We therefore also explored the role of another differentially abundant metabolite, TMAO, using the cancer cell-brain slice co-culture system (Figure 4A). However, TMAO did not alter the cancer cell-vessel interaction (Figures S4E and S4F), in contrast to KYNA, nor did it impact cancer cell growth *in vitro* (Figure S4G). We also evaluated the *in vivo* effect of TMAO administration in the drinking water using a similar schedule for the KYNA experiment (Figure 4G). We observed no difference in terms of BrM onset (Figure 4H), overall survival (Figure S4H), BrM progression (Figure S4I), and BrM number (Figure S4J) between TMAO-treated and control mice. However, we did find an increase in BrM volume in the TMAO treatment group compared to control (Figure S4K) though without an evident effect on survival (Figure S4H).

Collectively, our data indicate the regulation of breast-BrM initiation by the bacterial microbiome *in vivo*. We explored how the gut-to-brain axis was impacted by both antibiotic administration and at the BrM endpoint, assessing the gut microbiome composition and circulating metabolite abundance. Finally, we tested the functional role of KYNA *ex vivo* and *in vivo* as a microbiome-regulated molecule, partially modulating breast-BrM seeding.

## Discussion

Polymorphic microbes have been recognized as an enabling characteristic among the prototypical hallmarks of cancer^10^ and also exert functional roles in the onset and progression of various CNS disorders.^9^ Recent data have highlighted the involvement of the gut-to-brain axis in primary brain tumors.^11^^,^^12^ We were thus motivated to explore the potential contribution of the gut microbiome in metastatic brain tumors. We investigated an *in vivo* breast-BrM model, either housed under GF conditions or administered antibiotics, and thereby uncovered an association between the absence of the bacterial genus *Alistipes* and delayed BrM initiation. Additionally, we identified KYNA as a systemic metabolite differentially regulated by either antibiotic administration (enriched) or at the BrM endpoint (depleted) and revealed a functional role for KYNA in impeding cancer cell-vessel interactions in an *ex vivo* model of BrM seeding.

The use of GF mice represents the gold standard for elucidating the role of the bacterial microbiome *in vivo.*^20^ Our study incorporates the use of this experimental approach to investigate the bacterial microbiome in brain tumors for the first time. Earlier studies had reported accelerated primary brain tumor growth in mice following antibiotic treatment,^15^^,^^16^ but GF experiments were not performed. Collectively, these studies and our data herein suggest a functional impact of the gut microbiota in differentially modulating brain tumor biology in a disease-specific manner. Understanding the mechanisms through which the gut bacterial microbiome influences primary brain tumor growth and extending our findings to additional BrM types will illuminate differences and potential commonalities between primary and metastatic brain tumors.

We initially identified KYNA as a molecule potentially linking BrM seeding with the bacterial microbiome. KYNA has been described as neuromodulator, and regulation of its levels in the brain is reported to be independent of the systemic circulation.^25^ Here, we found a correlation between KYNA modulation and *Alistipes* abundance in the gut, suggesting a potential involvement of this bacterial genus in KYNA regulation. Addition of KYNA in brain slice cultures significantly reduced the interactions between BrM cells and the vasculature. By evaluating exogenous KYNA supplementation *in vivo*, we found a trend toward delayed BrM onset, indicating only a partial involvement of this molecule in the brain metastatic cascade under these experimental conditions. This is also consistent with the challenge for KYNA to effectively cross the BBB,^25^ even in the context of BrM, where we have reported that the BBB permeability is altered.^30^ Further experiments would be of interest to design strategies to enhance KYNA delivery in the brain and to assess the combination of KYNA with other metabolites/molecules impacted by the bacterial microbiome as an approach to control BrM at the systemic level using orally administered non-cytotoxic agents.

In conclusion, investigating how the bacterial microbiome evolves and regulates primary and secondary tumors may facilitate the development of effective systemic treatments for brain tumors. This is especially important due to the challenges associated with drug delivery and the immunosuppressive environment in the brain, which often limit the efficacy of conventional therapeutical strategies.^6^ Collectively, our data indicate an important role of the gut-to-brain axis in breast-BrM initiation.

### Limitations of the study

There are some limitations to our study. Firstly, our investigation into the impact of BrM on the gut-brain axis was conducted using the PyMT-BrM3 breast-BrM model alone. This decision was driven by the presence of aggressive extracranial metastases in the EO771-BrM model, which could potentially introduce confounding factors in the evaluation of the gut microbiome and metabolites. Although we identified *Alistipes* as a bacterial genus exhibiting differential abundance in the gut following antibiotic administration and at the BrM endpoint, we did not explore its functional role *in vivo* in the breast-BrM model in the current study, and this will be important to investigate in the future. Our data indicate only a modest effect in the control of BrM onset by administration of the single metabolite KYNA in the drinking water. This result can be interpreted as not fully recapitulating the potential multifactorial effect exerted by different agents, including several metabolites, that are regulated by the bacterial microbiome in the modulation of BrM onset. It may also reflect the technical challenges with administering metabolites *in vivo* in a manner that long-term stability and delivery to the target organs is assured. In summary, although our study provides valuable insights into the interplay between the bacterial microbiome, circulating metabolites, and breast-BrM initiation, future studies will be important to evaluate the potential translational relevance.

## Resource availability

### Lead contact

Further information and requests for resources and reagents should be directed to, and will be fulfilled by, the lead contact Prof. Johanna Joyce (johanna.joyce@unil.ch).

### Materials availability

PyMT-BrM3 GFP cells were generated for this study, and they are available upon request to the lead contact.

### Data and code availability

• Data reported in this paper will be shared by the lead contact upon request. 16S-seq data are deposited at the following repository GEO: https://www.ncbi.nlm.nih.gov/geo/query/acc.cgi?acc=GSE286123.
• This paper does not report original code.
• Any additional information required to reanalyze the data reported in this paper is available from the lead contact upon request.

## Acknowledgments

We thank all members of the Joyce lab for insightful comments and discussion. We are grateful to the *in vivo* and germ-free facilities at the University of Lausanne and the CHUV Lausanne for their support for antibiotic treatments and germ-free experiments. This research was supported in part by the 10.13039/100014599Mark Foundation for Cancer Research ASPIRE award, 10.13039/100001006Breast Cancer Research Foundation, 10.13039/100006352Ludwig Institute for Cancer Research, and 10.13039/501100006390University of Lausanne (to J.A.J.). M.M. gratefully acknowledges support for his postdoctoral fellowship jointly awarded from the Italian Association of Cancer Research (AIRC) and the 10.13039/100010665Marie Skłodowska-Curie grant agreement No. 800924. R.S.M. is supported by the 10.13039/501100007601European Union’s Horizon 2020 research and innovation program under the Sklodowska Curie grant agreement (no. 955951, Evomet ITN). G.M. is supported by the 10.13039/100000002National Institutes of Health/National Cancer Institute (NIH/NCI) F32 CA260769. J.A.W. acknowledges support from 10.13039/100000002NIH/NCI R01 CA219896 and the Stand Up to Cancer Convergence Grant. Experimental schematic images were generated using Biorender.

## Author contributions

M.M. and J.A.J. conceived the study, designed the experiments, and interpreted the data. M.M. performed all experiments and analyses with support for certain experiments from M.B., B.D., V.W., E.L., and R.S.M. The 16S-seq data were analyzed and interpreted by G.M., A.V.D., M.C.W., N.J.A., and J.A.W. S.C. helped organize the germ-free experiments. H.G.A. and J.I. supervised the acquisition and processing of metabolomic data and performed quality assessment. J.L., A.K., and N.F. analyzed the metabolomics data. M.M. prepared the figures. M.M. and J.A.J. wrote the manuscript. J.A.J. supervised the study. All authors reviewed, edited, or commented on the manuscript.

## Declaration of interests

M.M., M.B., B.D., G.M., V.W., E.L., J.L., S.C., H.G.-A., R.S.M, A.K., N.F. A.V.D, M.C.W, J.I., and N.J.A. declare no competing interests; M.M. at the time of publication is affiliated to the Institute of Oncology Research (IOR), Bellinzona 6500, Switzerland. J.A.J. has received an honorarium for speaking at a research symposium organized by Bristol Meyers Squibb and previously served on the scientific advisory board of Pionyr Immunotherapeutics (declarations for last 3 years). J.A.W. is an inventor of US patent applications WO2020150429A1, US20200129569A1, WO2019191390A2, and WO2020106983A1 and reports advisory roles and honoraria from Daiichi Sankyo, Gustave Roussy Cancer Center, EverImmune, and OSE Immunotherapeutics. J.A.W. receives stock options from Micronoma.

## STAR★Methods

### Key resources table

**Table:** 

REAGENT or RESOURCE	SOURCE	IDENTIFIER
**Antibodies**		

FCM: CD45 AF700, rat monoclonal anti-mouse (clone 30-F11), dilution 1:200	Biolegend	Cat# 103128; RRID:AB_493715
FCM: CD11b BUV661, rat monoclonal anti-mouse (clone M1/70), dilution 1:640	BD Biosciences	Cat# 612977; RRID:AB_2870249
FCM: Ly-6C BV711, rat monoclonal anti-mouse (clone HK1.4), dilution 1:800	Biolegend	Cat# 128037; RRID:AB_2562630
FCM: Ly-6G PE-Cy7, rat monoclonal anti-mouse (clone 1A8), dilution 1:300	Biolegend	Cat# 127618; RRID:AB_1877261
FCM: CD49d BV789, rat monoclonal anti-mouse (clone R1-2), dilution 1:160	BD Biosciences	Cat# 564397; RRID:AB_2738789
FCM: CD206 APC, rat monoclonal anti-mouse (clone C068C3), dilution 1:50	Biolegend	Cat# 141708; RRID:AB_10900231
FCM: I-A/I-E (MHCII) AF700, rat monoclonal anti-mouse (clone M5/114.15.2), dilution 1:800	Biolegend	Cat# 107621; RRID:AB_493726
FCM: CD4 BV650, rat monoclonal anti-mouse (clone GK1.5), dilution 1:200	BD Biosciences	Cat# 613006; RRID:AB_2870274
FCM: TCR β chain AF488, rat monoclonal anti-mouse (clone H57-597), dilution 1:250	Biolegend	Cat# 109215; RRID:AB_493344
FCM: CD19 PE, rat monoclonal anti-mouse (clone 6D5), dilution 1:500	Biolegend	Cat# 115508; RRID:AB_313643
FCM: CD8a PerCP/Cy5.5, rat monoclonal anti-mouse (clone 53–6.7), dilution 1:150	Biolegend	Cat# 100734; RRID:AB_2075238
FCM: TCR γ/δ chain BV421, rat monoclonal anti-mouse (clone GL3), dilution 1:200	Biolegend	Cat# 118120; RRID:AB_2562566
FCM: NK-1.1 BUV395, rat monoclonal anti-mouse (clone PK136), dilution 1:180	BD Biosciences	Cat# 564144; RRID:AB_2738618
IF: GFP, chicken polyclonal to GFP, dilution 1:1000	Abcam	Cat# ab13970; RRID:AB_300798
IF: CD31, rat monoclonal anti-mouse (clone MEC 13.3), dilution 1:300	BD Biosciences	Cat# 550274; RRID:AB_393571
IF: anti-chicken IgG (H + L) AF488, dilution 1:500	Jackson ImmunoResearch	Cat# 703-545-155; RRID:AB_2340375
IF: anti-rat IgG (H + L) AF647, dilution 1:500	Abcam	Cat# ab150155; RRID:AB_2813835

**Chemicals, peptides, and recombinant proteins**		

DMEM-F12 (1:1), GlutaMAX	Gibco, Thermo Fisher	Cat# 31331028
HBSS	Gibco, Thermo Fisher	Cat# 14175-095
Penicillin/Streptomycin	Gibco, Thermo Fisher	Cat# 15140122
Fetal Bovine Serum (FBS)	Gibco, Thermo Fisher	Cat# 10270-106
Trypsin-EDTA (0.05%), phenol red	Gibco, Thermo Fisher	Cat# 25300054
PBS	Gibco, Thermo Fisher	Cat# 20012027
Normal donkey serum	SigmaAldrich	Cat# S30-M
Triton X-100	Applied Chemicals	Cat# A4975
Fluorescence Mounting Medium	Dako, Agilent	Cat# S302380
Agarose low gelling temperature	Sigma,	Cat# A9414-100
Bovine Serum Albumin	Jackson ImmunoResearch	Cat# 001-000-162
UltraPure™ 0.5M EDTA	Invitrogen	Cat# 15575020
Pentobarbital	CHUV Hospital, Lausanne, Switzerland	N/A
Gadobutrol (Gadovist)	Bayer	N/A
Attane™ Isoflurane	Attane	N/A
Ketamine	Sintetica	N/A
Medetomidine	Vétoquinol Italia S.r.l.	N/A
Buprenorphine	Streuli	N/A
Atipamezole	Vétoquinol Italia S.r.l.	N/A
Ampicillin	Sigma-Merk	Cat# A9393
Neomycin trisulfate	Sigma-Merk	Cat# N1876
Metronidazole	Sigma-Merk	Cat# M1547
Vancomycin	Goldbio	Cat# V-200
Bacitracin	Sigma-Merk	Cat# 11702
Sucrose	Axonlab	Cat# A2211
KYNA	Sigma-Merk	Cat# K3375
TMAO	Sigma-Merk	Cat# 317594
DMSO	PanReac AppliChem	Cat# A3672
PFA	Electron Microscopy Sciences	Cat# 15714-S
Methanol	Sigma-Merk	Cat# 1.06035
Formic acid	Sigma-Merk	Cat# 5.33002
Glucose	ThermoFisher	Cat# A2494001
HEPES pH 7.4	ThermoFisher	Cat# 15630049

**Critical commercial assays**		

Zombie NIR Fixable Viability Kit	BioLegend	Cat# 423106
4′, 6- Diamidino-2-Phenylindole, Dihydrochloride (DAPI)	Life Technologies	Cat# D1306
Tumor Dissociation Kit, mouse	Miltenyi	Cat# 130-096-730
QIAamp PowerFecal Pro DNA kit	Qiagen	Cat# 51804

**Deposited data**		

16S data seq	This study	https://www.ncbi.nlm.nih.gov/geo/query/acc.cgi?acc=GSE286123

**Experimental models: Cell lines**		

MMTV-PyMT (murine mammary tumor virus; Polyoma middle T antigen); PyMT-BrM3	Croci et al.^19^ Prof. J.A. Joyce	Available upon request
MMTV-PyMT (murine mammary tumor virus; Polyoma middle T antigen); PyMT-BrM3-GFP	This study	Available upon request
EO771	ATCC	Cat# CRL-3461

**Experimental models: Organisms/strains**		

C57BL/6J mice	The Jackson Laboratory	RRID: IMSR_JAX:000664
Germ-free C57BL/6J mice, line 3002	Department of Biomedical Research of the University of Bern, Switzerland	N/A

**Oligonucleotides**		

341F	CosmosID	N/A
805R	CosmosID	N/A

**Recombinant DNA**		

Plasmid: GFP	Provided by prof. I. Malanchi	N/A

**Software and algorithms**		

FlowJo, v10.5.3	Tree Star	https://www.flowjo.com/
GraphPad Prism, v10.0.3	GraphPad Software	https://www.graphpad.com/scientific-software/prism/
ImageJ, v1.0	Open Source	https://imagej.net/ij/
Mass Hunter Quantitative analysis software	Agilent Technologies	https://www.agilent.com/en/product/software-informatics/mass-spectrometry-software/data-analysis/quantitative-analysis
R, v. 4.0.5	CRAN	https://cran.r-project.org/bin/windows/base/old/4.0.5/
DADA2 package, v1.16.0	Github	https://github.com/benjjneb/dada2/releases
Phyloseq package, v1.44.0	Bioconductor	https://www.bioconductor.org/packages/release/bioc/html/phyloseq.html
Decontam package, v1.20.0	Bioconductor	https://www.bioconductor.org/packages/release/bioc/html/decontam.html
ComplexHeatmap package	Bioconductor	https://bioconductor.org/packages/release/bioc/html/ComplexHeatmap.html
PMCMRplus package,v. 1.9.0	CRAN	https://cran.r-project.org/web/packages/PMCMRplus/index.html
ANCOM-BC package, v1.6.2	Bioconductor	https://www.bioconductor.org/packages/release/bioc/html/ANCOMBC.html
Vegan R package, v2.6-4	CRAN	https://cran.r-project.org/web/packages/vegan/index.html

**Other**		

gentleMACS Octo Dissociator	Miltenyi	Cat# 130-095-937
gentleMACS C Tubes	Miltenyi	Cat# 130-096-334
LS Columns	Miltenyi	Cat# 130-042-401
Fortessa flow cytometer	BD Bioscience	N/A
3T MRI	Bruker	N/A
Vibratome	Leica	Cat# VT1200
Ti2 spinning disk	Nikon	N/A
Mass spectrometer HILIC-MS/MS	Agilent Technologies	N/A
Mass spectrometer LC-HRMS,	ThermoFisher	N/A
Illumina Miseq platform 2x250bp	Illumina	N/A
Coverslip	ThermoFisher	Cat# BB02400500A113FST0
Microscope slide	Fisherbrand	Cat# 12-550-15
Vetbond tissue adhesive	3M	Cat# 1469SB
K2EDTA-coated tubes	Greiner	Cat# 450532
Nuclepore Track-Etched Polycarbonate PVP-Free (hydrophobic), 8.0 μm Pore Size	Cytivia	Cat# 150446

### Experimental model and study participant details

#### Cell lines

The PyMT-BrM3 cell line was generated as previously described.^19^ Briefly, the brain-homing capacity of a cell line originally isolated from a metastatic lymph node from an MMTV-PyMT mouse was enriched via three rounds of *in vivo* selection and *in vitro* expansion. EO771 cells were obtained from the ATCC. PyMT-BrM3-GFP cells were generated using a second-generation lentiviral system transducing a GFP plasmid (kindly provided by Prof. Ilaria Malanchi) generated by cloning an empty pENTRY donor vector and a lentiviral destination vector (cPPT-CMVenh-attR-mPGK-GFP-WPRE on a pRRL backbone) via the LR Gateway system. This resulted in a lentivirus plasmid expressing GFP only as an ORF, under the control of the mPGK promoter. All cell lines were maintained in DMEM-F12 media (Gibco, ThermoFisher, cat. no. 31331028) supplemented with 10% FBS (Gibco, ThermoFisher, cat. no. 10270106), and 1% penicillin/streptomycin (P/S) (P/S, Gibco, ThermoFisher, cat. no. 15140122).

#### Animals

Wild-type C57BL/6J mice were bred within the University of Lausanne animal facilities. Germ-free (GF) C57BL/6J mice (line 3002) were obtained from the Department of Biomedical Research of the University of Bern, Switzerland. They were bred and maintained in the germ-free animal facility at the Center Hospitalier Universitaire Vaudois (CHUV Lausanne) at 30–50% relative humidity, 23 ± 2°C and 12-h light/dark cycles. Autoclaved tap water and diet (D131, Safe) were provided *ad libitum*. Mice were housed with environmental enrichment. All animal studies were first approved by the Institutional Animal Care and Use Committees of the University of Lausanne and Canton Vaud, Switzerland under licenses VD3314 and VD3688.

#### Brain metastasis *in vivo* models and treatments

To generate breast-BrM, 6- to 10-week-old females were injected in the left cardiac ventricle with 1x10^5^ PyMT-BrM3 cells resuspended in 100 μL HBSS (Gibco, ThermoFisher, cat. no. 14175-095). Cell injection was performed under isoflurane anesthesia (O_2_ + 2% isoflurane) for the antibiotic treatment experiments. For GF mice and corresponding controls, mice were anesthetized with a mix of Ketamine (75 mg/kg; Sintetica), Medetomidine (0.5 mg/kg; Domitor, Vétoquinol Italia S.r.l.), and Buprenorphine (0.05 mg/kg; Streuli). The anesthetic effect was reserved using Atipamezole (1 mg/kg; Antiseda, Vétoquinol Italia S.r.l.). To detect BrM, mice were imaged by 3T MRI (Bruker), following intraperitoneal (i.p.) injection with 150 μL gadobutrol (Gadovist, 1 mmol mL^−1^, Bayer). Mice were sacrificed at the predefined humane endpoint of the experiment. For antibiotic treatment, mice were treated via the drinking water with a broad-spectrum cocktail (4-ABX) including Ampicillin (1 g/L; Sigma-Merk, cat. no. A9393), Neomycin trisulfate (1 g/L; Sigma-Merk, cat. no. N1876), Metronidazole (1 g/L, Sigma-Merk, cat. no. M1547), and Vancomycin (0.5 g/L; Goldbio, cat. no. V-200) in sucrose (2%; Axonlab, cat. no. A2211) or the selective cocktail (2-ABX) including Bacitracin (1 g/L; Sigma-Merk cat. no. 11702), and Neomycin trisulfate (1 g/L) in sucrose (2%), or the CTRL group of sucrose alone (2%) as indicated in the experimental description. For KYNA treatment, mice were treated via the drinking water with KYNA (25 mg/L, Sigma-Merk, cat. no. K3375). For TMAO treatment, mice were treated via the drinking water with TMAO (1 g/L, Sigma-Merk, cat. no. 317594).

#### Human samples/clinical trials

This study does not include any human samples or data from clinical trials.

### Method details

#### Flow cytometry

Mice were euthanized by terminal anesthesia using pentobarbital (Lausanne University Hospital, CHUV), followed by transcardial perfusion with PBS (Gibco, ThermoFisher, cat. no. 20012027). Tissue was digested using a Tumor Dissociation Kit (Miltenyi, cat. no. 130-096-730) following the manufacturer’s protocol. The cell suspension was filtered using a 100 μm filter and washed with FACS buffer (PBS, 0.5% BSA (Jackson ImmunoResearch, cat. no. 001-000-162), 2mM EDTA (Thermo Fischer Scientific, cat. no. 15575020)). To exclude dead cells from analysis, cells were stained with Near Far-Red Zombie (BioLegend, cat. no. 423106) in PBS. The single-cell suspension was stained with antibodies listed in Table S3. All antibodies were purchased from BD Bioscience and BioLegend. Flow cytometry data were acquired using an LSR Fortessa (BD Bioscience), and data were analyzed with FlowJo Software v10.5.3 (Tree Star).

#### 16S rRNA gene sequencing

Fecal samples from a single mouse were collected in sterile tubes directly from the rectum of mice and stored at −80°C. DNA was extracted using the QIAamp PowerFecal Pro DNA kit (Qiagen, cat. no. 51804) following the manufacturer’s protocol in a clean environment using autoclaved tools. 16S sequencing was performed by an external company (CosmosID) starting from 5 ng of genomic DNA. Libraries were constructed by amplification via PCR with 341F and 805R primers covering the V3-V4 region. Sequencing was performed using the Illumina Miseq platform 2x250bp. The 16S rRNA gene sequencing datasets were processed using the DADA2 package (version 1.16.0).^31^ Files were imported into R using the phyloseq package (Version 1.44.0).^32^ The data was processed using the decontam package (Version 1.20.0),^33^ using the isContaminant function with a 0.5 threshold and the environmental control used as a negative control. After removal of environmental controls, data was rarefied. Alpha diversity was calculated with the phyloseq package using the Shannon index. Heatmaps were generated using the ComplexHeatmap package.^34^

#### Metabolomics analysis

Plasma was isolated from whole blood collected by intracardiac puncture between 9a.m. and 11a.m. from terminally anesthetized mice in K2EDTA-coated tubes (Greiner, cat. no. 450532) and centrifuged twice for 10 min at 10000 g at 4°C, moving each supernatant to a new tube after each centrifugation. Finally, the plasma was stored at −80°C. Bile acids and high-coverage targeted analysis of polar metabolites were performed at the Metabolomics Platform of the University of Lausanne as previously described.^35^^,^^36^^,^^37^^,^^38^ Briefly, for bile acid analysis, plasma aliquots (25 μL) were mixed with 100 μL of the ice-cold internal standard solution (in 100% MeOH), and 600 μL of H_2_O with 0.2% formic acid, loaded onto solid phase extraction plates and further processed for the phospholipid removal and bile acids pre-concentration. Quantification was performed using a Liquid chromatography-high-resolution mass spectrometry system (LC-HRMS using Vanquish Horizon UHPLC coupled with Q-Exactive Focus mass spectrometer interfaced with a HESI source, ThermoFisher Scientific) operating in negative ionization mode. Chromatographic separation was performed as previously described.^39^ The internal standard mixture contained 13 deuterium-labeled bile acids (Table S4). For high-coverage targeted analysis of polar metabolites, plasma (20μL) was extracted with the addition of ice-cold methanol (80 μL), vortexed and centrifuged for 15 min at 15000 g, at 4°C. The supernatants were transferred to LC-MS vials for injection. They were analyzed by hydrophilic interaction chromatography coupled to tandem mass spectrometer (HILIC-MS/MS using 6496 iFunnel triple quadrupole mass spectrometer interfaced with 1290 UHPLC system, Agilent Technologies) operating in multiple reaction monitoring (MRM) in both positive and negative ionization modes, to maximize the polar metabolome coverage.^40^ Raw data were processed using the Mass Hunter Quantitative analysis software (Agilent Technologies).

#### *Ex vivo* brain metastasis seeding assays

Organotypic co-cultures of brain slices and cancer cells were performed as previously described.^41^ Briefly, brains from 6- to 10-week-old females were isolated after terminal anesthesia using pentobarbital (Lausanne University Hospital, CHUV), followed by transcardial perfusion with brain dissection media (HBSS with calcium and magnesium (Gibco, ThermoFisher, cat. no. 14025-092), 30 mM glucose (Gibco, ThermoFisher, cat. no. A2494001), 2.5 mM HEPES pH 7.4 (Gibco, ThermoFisher, cat. no. 15630049), 1% P/S. Brains were then embedded in 4% agarose low gelling temperature (Sigma, cat. no. A9414-100), cut using a vibratome (Leica) with thickness 250 μm, speed 0.3 mm/s, and amplitude 1 μm, and temporarily collected in brain dissection media. Brain slices were then cut in half and transferred into a 12 well-plate using a flat spatula on a 0.8 μm pore membrane (Cytivia, cat. no. 150446) floating in complete media (DMEM-F12, 10% FCS, 1% P/S) with either vehicle (DMSO; PanReac AppliChem cat. no. A3672), KYNA 10 μM (Sigma-Merck, cat. no. K3375), or TMAO 10 μM (Sigma-Merck, cat no. 317594). After 2 h of incubation at 37°C, 5% CO_2_, brain slices were removed from the plate by lifting the membrane, and 3x10^4^ PyMT-BrM3-GFP cells resuspended in 2 μL of HBSS were seeded on top. After 1 min, brain slices were placed again in the media. After 4 days, slices were fixed with 4% PFA (Electron Microscopy Sciences, cat. no. 15714-S) overnight, then washed three times with PBS (Gibco, ThermoFisher, cat. no. 14190-136). To prepare samples for IF staining, tissues were permeabilized and blocked with 0.25% Triton X-100 (PanReac AppliChem, cat. No. A4975) in PBS and 5% donkey serum (EMD Millipore, Merk, cat. No. S30-M) in PBS for 2 h at room temperature, followed by incubation with primary antibodies listed in Table S5 in 5% donkey serum overnight at 4°C. Secondary staining was performed in 5% donkey serum with antibodies listed in Table S5 for 2 h at room temperature. Slides were washed three times with PBS and counterstained with DAPI for 30 min. Finally, slides were washed and mounted with mounting media (Dako, cat. no. S302380) and a coverslip (Menzel-Gläser, Thermo Scientific, cat. no. 631–0973). The entire area of stained brain slices was imaged with a Nikon Ti2 spinning disk microscope (Nikon) acquiring a 16 μm-thick volume in 3 z stacks. Maximum intensity projection images were analyzed using ImageJ v1.0.

### Quantification and statistical analysis

Prism 9 was used to perform statistical analysis and graphically plot all data exception for metabolomic data. Data were tested for normal distribution with the Shapiro-Wilk test. Normally distributed data were analyzed by a paired or unpaired two-tailed Student’s t test. Non-normally distributed data were analyzed by a paired or unpaired two-tailed Student’s t test with Mann-Whitney correction. Survival curves were compared using Mantel-Cox log-rank test. In figures presenting pooled data, data are represented as mean ± SD. ∗, *p* < 0.05; ∗∗, *p* < 0.01; ∗∗∗, *p* < 0.001; ∗∗∗∗, *p* < 0.0001; ns: non-significant.

For bile acid analysis, values under the detection limit were replaced by the technical lower abundance detected for each metabolite divided by two (Table S2). Then, bile acid concentrations were normalized by log transformation and Pareto scaling of the data. In the case of polar metabolites, we used variance stabilizing normalization. Normalization was performed using custom R functions. For the differential analysis between CTRL, 4-ABX, and 2-ABX, bile acids were identified as differentially abundant metabolites by first applying a Kruskal-Wallis rank sum non-parametric test followed by a Dunn’s multiple comparison non-parametric test. For polar metabolites, we first performed a one-way ANOVA analysis followed by Tukey’s multiple pairwise-comparisons tests. For downstream analysis, we selected metabolites in which at least one of the treatments was significantly different from the control. Concerning the differential analysis between CTRL and BrM endpoint, bile acids were identified as differentially regulated metabolites using a Wilcoxon non-parametric test. Polar metabolite data were analyzed using an unpaired two-tailed Student’s t test. R and the packages stats (v. 4.0.5) and PMCMRplus (v. 1.9.0) were used to analyze the metabolomic data. *p* values <0.05 were considered statistically significant. For these analyses, any missing values were replaced by the metabolite-specific limit of detection divided by two.

For the gut microbiome composition analysis, a Kruskal-Wallis test was used for comparison between control, 4-ABX and 2-ABX groups, and the Analysis of Compositions of Microbiomes with Bias Correction (ANCOM-BC R package, version 1.6.2) was used for comparing control and BrM endpoint.^42^ Adjusted *p* values were calculated using the Holm method and q values <0.05 were considered statistically significant. For beta diversity comparison, the adonis2 test was used (Vegan R package version 2.6–4). Each specific statistical test used is reported for each experiment in the figure legends.

## Supplemental information

Document S1. Figures S1–S4 and Tables S3–S5

Table S1. Bacterial quantification in fecal samples, related to Figures 2 and S2 Individual excel sheet reporting the relative bacteria abundance in fecal material generated by 16S rRNA sequencing.

Table S2. Metabolite quantification in plasma, related to Figures 3 and S3 Individual excel sheet reporting the absolute bile acid and relative polar metabolite abundance in plasma generated by mass spectrometry.
