# Supplementary material for: The bacterial microbiome modulates the initiation of brain metastasis by impacting the gut-to-brain axis
Source: iScience. 2025 Jan 22;28(2):111874. doi: 10.1016/j.isci.2025.111874 (PMC11848439; doi:10.1016/j.isci.2025.111874)
Supplement: Document S1. Figures S1–S4 and Tables S3–S5 [file mmc1.pdf]

iScience, Volume *28*

Supplemental information

The bacterial microbiome modulates the initiation of brain metastasis by impacting the gut-to-brain axis

Matteo Massara, Michelle Ballabio, Bastien Dolfi, Golnaz Morad, Vladimir Wischnewski, Eleni Lamprou, Joao Lourenco, Stéphanie Claudinot, Hector Gallart-Ayala, Rui Santalla Méndez, Annamaria Kauzlaric, Nadine Fournier, Ashish V. Damania, Matthew C. Wong, Julijana Ivanisevic, Nadim J. Ajami, Jennifer A. Wargo, and Johanna A. Joyce

Figure S1

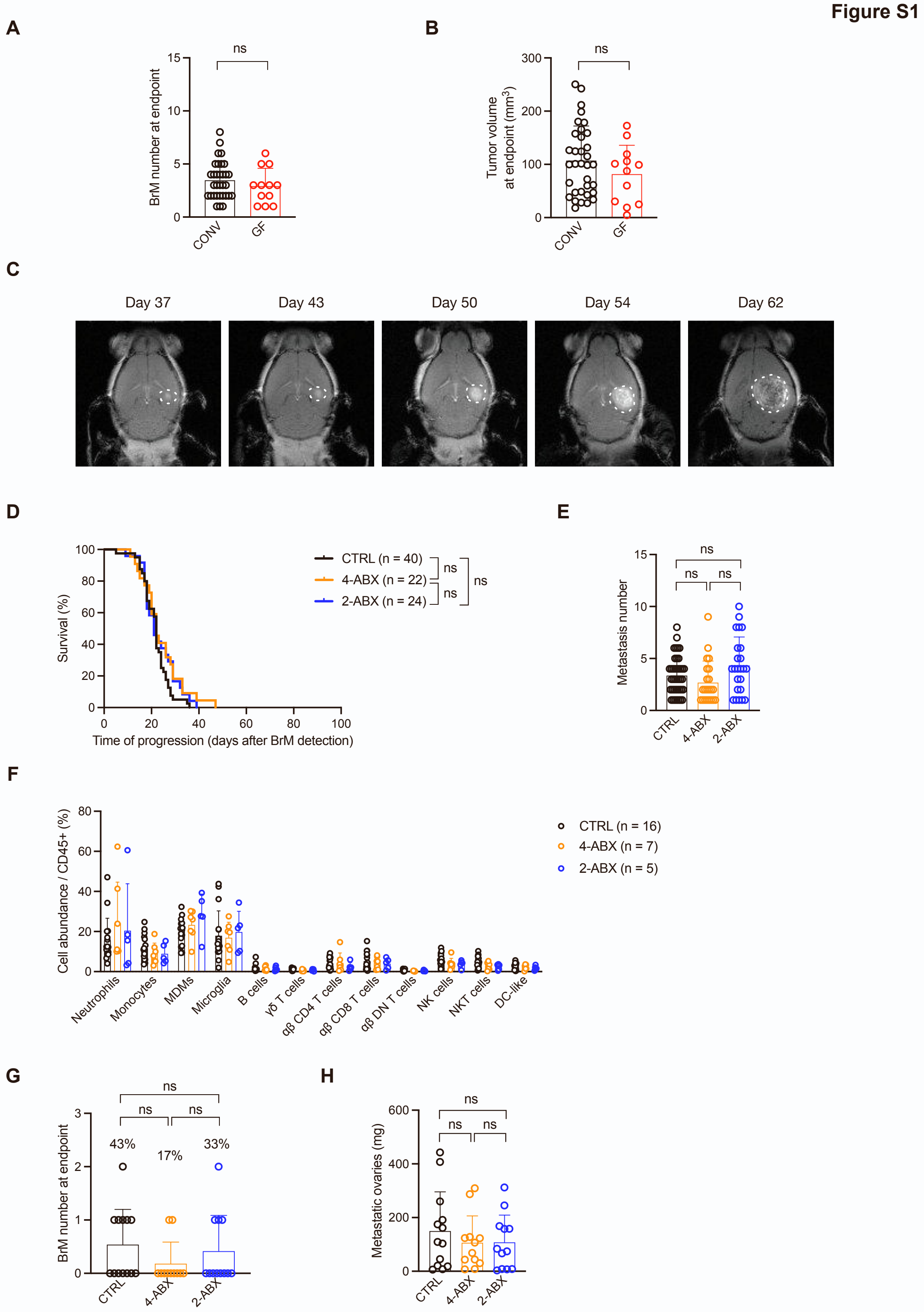

A
BrM number at endpoint
ns
CONV
GF
B
Tumor volume at endpoint (mm3)
ns
CONV
GF
C
Day 37
Day 43
Day 50
Day 54
Day 62
D
Survival (%)
Time of progression (days after BrM detection)
CTRL (n = 40)
4-ABX (n = 22)
2-ABX (n = 24)
ns
E
Metastasis number
ns
CTRL
4-ABX
2-ABX
F
Cell abundance / CD45+ (%)
CTRL (n = 16)
4-ABX (n = 7)
2-ABX (n = 5)
Neutrophils
Monocytes
MDMs
Microglia
B cells
γδ T cells
αβ CD4 T cells
αβ CD8 T cells
αβ DN T cells
NK cells
NKT cells
DC-like
G
BrM number at endpoint
ns
43%
17%
33%
CTRL
4-ABX
2-ABX
H
Metastatic ovaries (mg)
ns
CTRL
4-ABX
2-ABX

Supplementary Figure 1. Microbiome manipulation does not impact BrM volume or number at the BrM endpoint, Related to Figure 1

(A) BrM number at endpoint in the PyMT-BrM3 model housed either in conventional (CONV) or germ-free (GF) animal facilities. **(B)** Tumor volume at the endpoint in the PyMT-BrM3 model housed in CONV or GF facilities. For (A, B), n = 34 for CONV, n=12 for GF; sum of three independent experiments. **(C)** Representative images of BrM in the PyMT-BrM3 model from a single mouse followed longitudinally by MRI. The BrM is encircled by a white dotted line. **(D)** Kaplan-Meier curves showing BrM progression (days after BrM detection) in CTRL, 4-ABX, and 2-ABX treated mice. **(E)** BrM number at the endpoint of mice in the CTRL, 4-ABX, or 2-ABX groups. **(F)** Relative immune cell composition on total CD45+ events of BrM lesions at the endpoint measured by flow cytometry. n = 16 for CTRL; n = 7 for 4-ABX and n = 5 for 2-ABX. **(G)** BrM number at endpoint in the EO771 model of mice treated either with CTRL, 4-ABX, or 2-ABX. The percentage of BrM penetrance is reported on the graph. **(H)** Metastatic ovary weight at the endpoint in the EO771 model in CTRL, 4-ABX, or 2-ABX groups. For (G and H), n = 13 for CTRL, n = 12 for 4-ABX, n = 12 for 2-ABX; single experiment. For (H), each data point represents the sum of two ovaries from a single mouse. Statistical analysis in (A) was performed using the unpaired t-test with Mann-Whitney correction. Statistical analysis in (B) was performed using the unpaired t-test. Statistical analysis in (D) was performed with the Mantel-Cox log-rank test. Statistical analysis in (E, G, and H) was performed using Kruskal-Wallis test. Statistical analysis in (F) was performed using two-way ANOVA and no statistically significant differences were observed. Data are represented as mean ± SD. ns: non-significant.

Figure S2

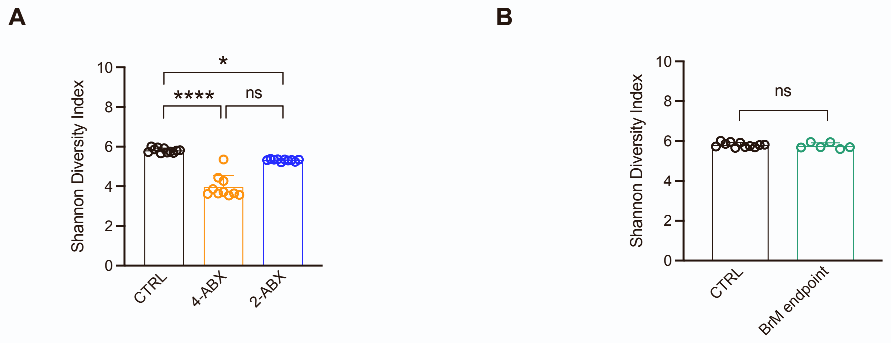

Supplementary Figure 2. Assessment of bacterial diversity after antibiotic administration and at the BrM endpoint, Related to Figure 2

(A) Shannon diversity index depicting the gut bacteria composition from healthy mice treated with either CTRL, 4-ABX, or 2-ABX, assessed at the bacterial genus level. n = 11 for CTRL, n = 10 for both 4-ABX and 2-ABX groups. **(B)** Shannon diversity index of gut bacteria composition from CTRL and BrM endpoint mice, analyzed at the bacterial genus level. n = 11 for CTRL; n = 6 for BrM endpoint. Statistical analysis in (A) was performed using Kruskal-Wallis test. Statistical analysis in (B) was performed using unpaired t-test. Data are represented as mean ± SD. *, $p < 0.05$; ****, $p < 0.0001$; ns: non-significant.

Figure S3

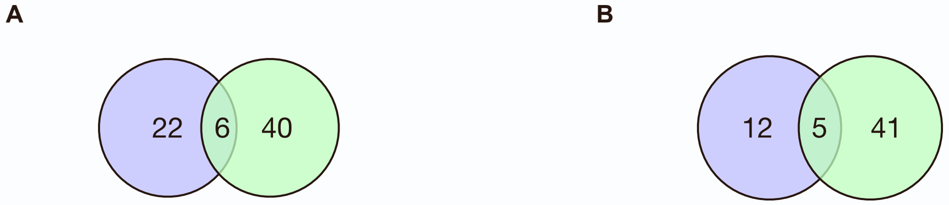

Supplementary Figure 3. Circulating metabolites commonly impacted by antibiotics and at BrM endpoint, Related to Figure 3

(A) Venn diagram depicting circulating metabolites that were significantly differentially abundant upon 4-ABX or 2-ABX treatments (blue circle) or at BrM endpoint (green circle).

(B) Venn diagram depicting circulating metabolites whose levels significantly increased or decreased upon 4-ABX treatment (blue circle) and at BrM endpoint (green circle).

Figure S4

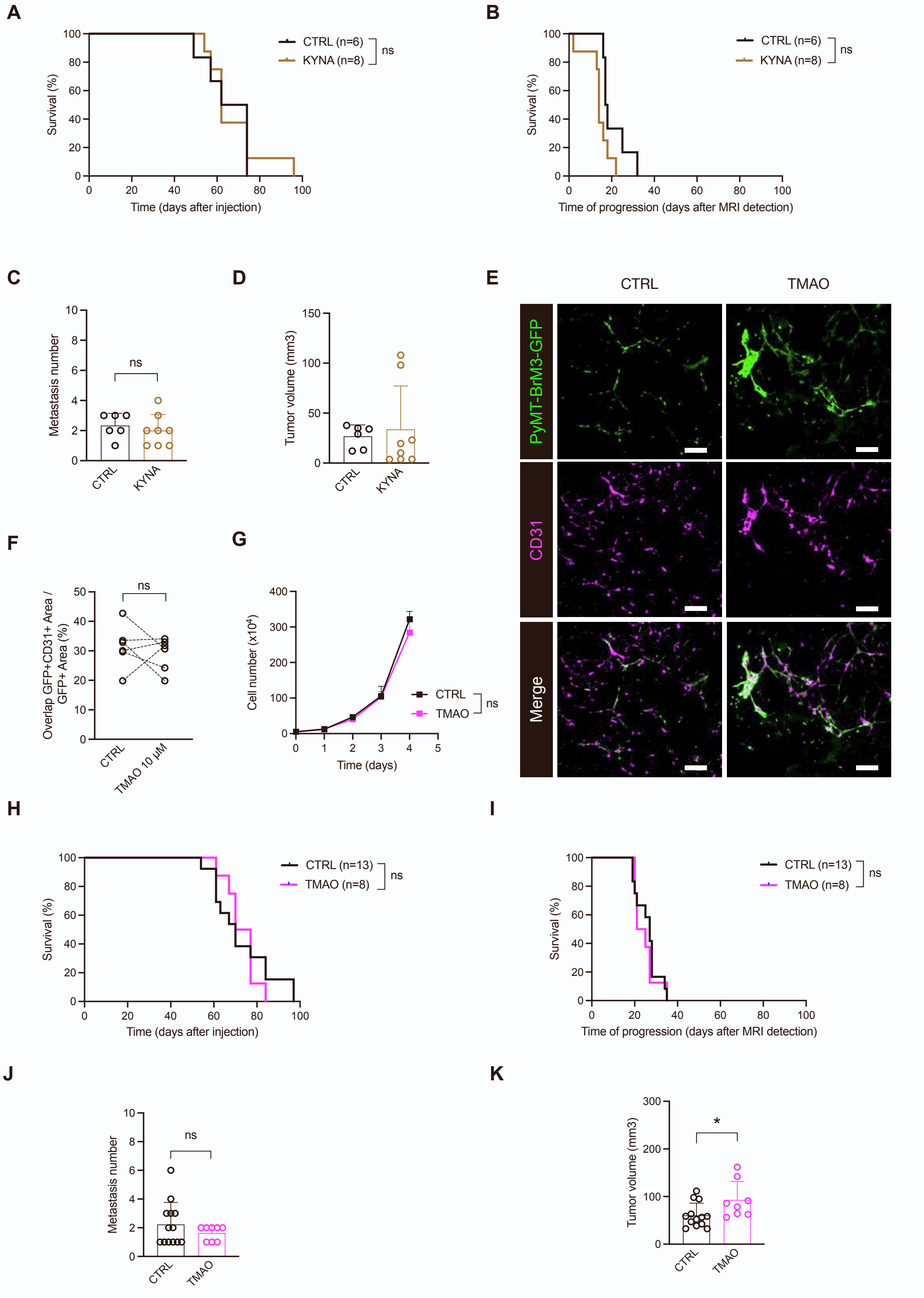

A
CTRL (n=6)
KYNA (n=8)
ns
Survival (%)
Time (days after injection)
B
CTRL (n=6)
KYNA (n=8)
ns
Survival (%)
Time of progression (days after MRI detection)
C
Metastasis number
ns
CTRL
KYNA
D
Tumor volume (mm3)
CTRL
KYNA
E
CTRL
TMAO
PyMT-BrM3-GFP
CD31
Merge
F
Overlap GFP+CD31+ Area / GFP+ Area (%)
ns
CTRL
TMAO 10 uM
G
Cell number (x10⁴)
CTRL
TMAO
ns
Time (days)
H
CTRL (n=13)
TMAO (n=8)
ns
Survival (%)
Time (days after injection)
I
CTRL (n=13)
TMAO (n=8)
ns
Survival (%)
Time of progression (days after MRI detection)
J
Metastasis number
ns
CTRL
TMAO
K
Tumor volume (mm3)
*
CTRL
TMAO

Supplementary Figure 4. TMAO and KYNA administration does not alter survival *in vivo* and TMAO does not interfere with cancer cell-vessel interaction *ex vivo*, Related to Figure 4

(A, B) Kaplan-Meier curves showing (A) the overall survival and (B) the BrM progression of CTRL and KYNA treated mice. **(C)** BrM number at the endpoint of mice in the CTRL and KYNA groups. **(D)** Tumor volume at the endpoint of mice in the CTRL and KYNA groups. For (A, B, C, D), n = 6 for CTRL, n = 8 for KYNA, single experiment. **(E)** Representative IF images of PyMT-BrM3 cells (green) and CD31 (magenta) in *ex vivo* brain slices after four days of incubation with CTRL or TMAO. Scale bar 200 μm. **(F)** Quantification of tissue area co-stained for GFP and CD31 as a proportion of the total GFP+ area. n = 6 mice. Sum of two independent experiments. **(G)** Growth curve of PyMT-BrM3 cells *in vitro* incubated in the presence of TMAO or CTRL. n = 3 per time point. Experiment was repeated twice, one representative experiment shown. **(H, I)** Kaplan-Meier curves showing (H) the overall survival and (I) the BrM progression of CTRL and TMAO treated mice. **(J)** BrM number at the endpoint of mice in the CTRL and TMAO groups. **(K)** Tumor volume at the endpoint of mice in the CTRL and TMAO groups. For (H, I, J, K), n = 13 for CTRL, n = 8 for TMAO, single experiment. Statistical analysis in (A, B, H, and I) was performed using the Mantel-Cox log-rank test. Statistical analysis in (C, D, J, and K) was performed using the t-test analysis. Statistical analysis in (F) was performed using the paired t-test. Statistical analysis in (G) was performed using two-way ANOVA. Data are represented as mean ± SD. *, $p < 0.05$; ns: non-significant.

Supplementary Tables

Table S3. Antibody list for flow cytometry analysis, Related to STAR Methods.
List of antibodies, clone number, manufacturer details and working dilution.

Antibody	Fluorophore	Clone	Manufacturer	Catalog number	Dilution
CD45	AF700	30-F11	BioLegend	103128	1:200
CD11b	BUV661	M1/70	BD Bioscience	612977	1:640
Ly-6C	BV711	HK1.4	BioLegend	128037	1:800
Ly-6G	PE-Cy7	1A8	BioLegend	127618	1:300
CD49D	BV789	R1-2	BD Bioscience	564397	1:160
CD206	APC	C068C2	BioLegend	141708	1:50
MHCII (I-A/I-E)	BV510	M5/114.15.2	BioLegend	107636	1:700
CD4	BV650	GK1.5	BD Bioscience	613006	1:200
TCRb	AF488	H57-597	BioLegend	109215	1:250
CD19	PE	6D5	BioLegend	115508	1:500
CD8a	PerCP/Cy5.5	53-6.7	BioLegend	100734	1:150
TCR g/d	BV421	GL3	BioLegend	118120	1:200
NK1.1	BUV395	PK136	BD Bioscience	564144	1:180
Zombie NIR	Near infrared		BioLegend	423106	1:200

Table S4. Internal standard mixture list for bile acid analysis, Related to STAR Methods. Table reporting the mass-to-charge (m/z) ratio, the internal standards, and retention times of bile acid standards.

**Bile Acid**	**Precursor m/z ratio**	**Internal Standard**	**Retention time (min)**
Lithocholic acid	375.29047	d4-LCA	20.6
7-Ketolithocholic acid	389.26973	d4-GDCA	15.5
Murocholic acid	391.28538	d4-CA	12.4
Ursodeoxycholic acid	391.28538	d4-GDCA	13.3
Chenodeoxycholic acid	391.28538	d4-CDCA	18.3
Deoxycholic acid	391.28538	d4-DCA	18.5
Isodeoxycholic acid	391.28538	d4-GDCA	19.7
3-Oxocholic acid	405.26465	d4-TCDCA	12.6
7-Ketodeoxycholic acid	405.26465	d4-GCA	11
α-Muricholic acid	407.2803	d4-GCA	10.8
β-Muricholic acid	407.2803	d4-GCA	11.2
ω-Muricholic acid	407.2803	d4-GCA	10.4
γ-Muricholic acid	407.2803	d4-GCA	11.8
Cholic acid	407.2803	d4-CA	12.6
Glycolithocholic acid	432.31193	d4-GDCA	13.5
Glycoursodeoxycholic acid	448.30685	d4-GCA	10.2
Glycohyodeoxycholic acid	448.30685	d4-CA	10.6
Glycochenodeoxycholic acid	448.30685	d4-GCDCA	12.9
Glycocholic acid	464.30176	d4-GCA	10.3
Taurolithocholic acid	482.29457	d5-TLCA	13
Tauroursodeoxycholic acid	498.28948	d4-TCA	5.1
Taurohyodeoxycholic acid	498.28948	d4-TCDCA	5.5
Taurochenodeoxycholic acid	498.28948	d4-TCDCA	10.6
Taurodeoxycholic acid	498.28948	d5-TDCA	10.9
Tauro-a-muricholic acid	514.2844	d4-TαMCA	2.1
Tauro-β-muricholic acid	514.2844	d4-TβMCA	2.3
Taurocholic acid	514.2844	d4-TCA	5.8

Table S5. Antibody list for immunofluorescence (IF) staining, Related to STAR Methods. List of antibodies, clone number, manufacturer details and working dilution.

**Antibody**	**Species**	**Clone**	**Manufacturer**	**Catalog number**	**Dilution**
GFP	Chicken	Polyclonal	Abcam	ab13970	1:500
CD31	Rat	MEC 13.3	BD Bioscience	550274	1:300
Anti-chicken IgG (H+L) Alexa Fluor 488	Donkey	Polyclonal	Jackson ImmunoResearch	703-545-155	1:500
Anti-rat IgG (H+L) Alexa Fluor 647	Donkey	Polyclonal	Abcam	ab150155	1:500
DAPI			Invitrogen	D1306	1:5000
